# Biomechanical comparison of locking plate and pin-tension band wiring fixation for 3D-printed canine patellar fracture repair

**DOI:** 10.3389/fvets.2025.1639433

**Published:** 2025-08-25

**Authors:** Seung-Gi Jung, Hwi-Yool Kim

**Affiliations:** Department of Veterinary Surgery, Graduate School of Veterinary Medicine, Konkuk University, Seoul, Republic of Korea

**Keywords:** canine patellar fracture, patellar locking plate, figure of eight pattern, tension band wiring, patellar transverse fracture repair

## Abstract

**Introduction:**

The conventional pin and tension band wiring (TBW) technique remains the standard for fixation, but is frequently associated with complications such as wire breakage, loosening, and delayed healing in patellar fracture. Locking plate fixation has demonstrated superior biomechanical stability in human studies. This study aimed to compare the biomechanical performance of locking plate fixation versus TBW in canine transverse patellar fractures and to evaluate the influence of plate design on fixation strength.

**Methods:**

Thirty 3D-printed canine patellar fracture models were fabricated based on CT data from a 45 kg Akita dog and allocated into three groups (*n* = 10 per group): Group 1—pin/TBW fixation, Group 2—2-hole locking plate fixation, Group 3—4-hole locking plate fixation. All models were subjected to tensile testing at a 135° stifle angle to simulate quadriceps force. Fixation failure was defined as a fracture gap displacement greater than 2 mm or structural yielding.

**Results:**

Group 1 showed progressive displacement with increasing tensile load (1 mm: 226.4 ± 26.2 N; 2 mm: 280.8 ± 27.7 N; 3 mm: 342.7 ± 27.0 N). Groups 2 and 3 exhibited less than 1 mm displacement and significantly higher maximum failure loads (Group 2: 505.6 ± 66.6 N; Group 3: 556.9 ± 39.6 N; *p* < 0.05). No significant difference was observed between the 2-hole and 4-hole plate groups.

**Discussion:**

Locking plate fixation demonstrated significantly superior biomechanical stability compared to the traditional pin/TBW technique in a canine transverse patellar fracture model. The comparable performance of the smaller 2-hole locking plate suggests its potential utility in clinical applications, particularly for small-breed dogs. These findings support the clinical applicability of locking plate systems as a reliable alternative for patellar fracture stabilization in veterinary practice.

## Introduction

The patella is a sesamoid bone located between the quadriceps femoris muscle group and the patellar ligament. It is the largest sesamoid bone in dogs and plays a critical role in efficiently facilitating the extension mechanism of the hindlimb (1). Although the patella is subjected to substantial tensile forces from the quadriceps, traumatic patellar fractures are rare in both veterinary and human medicine, accounting for ~0.1% of all fractures in dogs (2, 3).

Recent studies have also reported patellar fractures as postoperative complications following surgical correction of patellar luxation and cranial cruciate ligament rupture (CCLR) in dogs (3). patellar fracture has been reported as a rare complication after tibial plateau leveling osteotomy (TPLO), with an incidence ranging from 0.09 to 2% (4–8). As TPLO and similar procedures for correcting cranial cruciate ligament rupture are among the most frequently performed orthopedic surgeries in the hindlimb of dogs, the incidence of secondary patellar fractures is expected to rise.

In veterinary medicine, most patellar fractures are classified as transverse fractures, which are primarily caused by the strong tensile force of the quadriceps muscle acting above and below the fracture line. This mechanism is especially common when the animal falls and lands with the knee flexed. During landing, the knee joint is extended, the quadriceps contract accordingly, and the hindlimb touches the ground, resulting in abrupt bending of the stifle joint (9).

When a patellar fracture occurs, the extension mechanism becomes dysfunctional. The strong tensile force generated by the quadriceps muscle can lead to high complication rates, including implant failure (such as breakage or loosening), re-fracture, delayed union, and non-union (8, 10, 11).

In the surgical management of patellar fractures, veterinary medicine has adapted various fixation techniques based on biomechanical and clinical studies from human medicine. The most commonly recommended technique for patellar fracture repair in both humans and dogs is the use of pins and figure-of-eight tension band wiring (TBW) (12–14).

However, despite its long-standing status as the standard method, TBW has been frequently associated with complications such as wire breakage, loosening, and soft tissue irritation. These issues often result in delayed healing, fixation failure, and the need for revision surgery (15–17).

Recent studies suggest that locking plate fixation may yield superior outcomes compared to TBW (18, 19). In particular, biomechanical studies using human cadavers have demonstrated that fixation with a locking plate provides significantly stronger mechanical stability than TBW. Furthermore, clinical applications in veterinary patients have reported enhanced fixation strength, reduced postoperative discomfort, and lower complication rates, indicating the potential for faster recovery and improved rehabilitation following surgery.

The objective of this study was to evaluate whether fixation using a locking plate alone provides a statistically significant improvement in mechanical stability compared to TBW and to assess its clinical applicability for the treatment of transverse patellar fractures in dogs. Moreover, this study aimed to compare differences in fixation strength according to the design and the number of holes in the locking plate.

## Materials and methods

### Subject preparation

For this study, a right femur and patella model were fabricated using 3D printing with polycarbonate material, based on the anatomy of a 45 kg, 1.5-year-old male Akita dog with no abnormalities in the hindlimb joints or bones. The 3D printing was performed by Mameall, Inc. (Hwaseong-si, Gyeonggi-do, Republic of Korea). Biomechanical testing was conducted using a Universal Testing Machine at Daegu Gyeongbuk Institute of Science and Technology (DGIST, Daegu, Republic of Korea).

**Figure 1:**
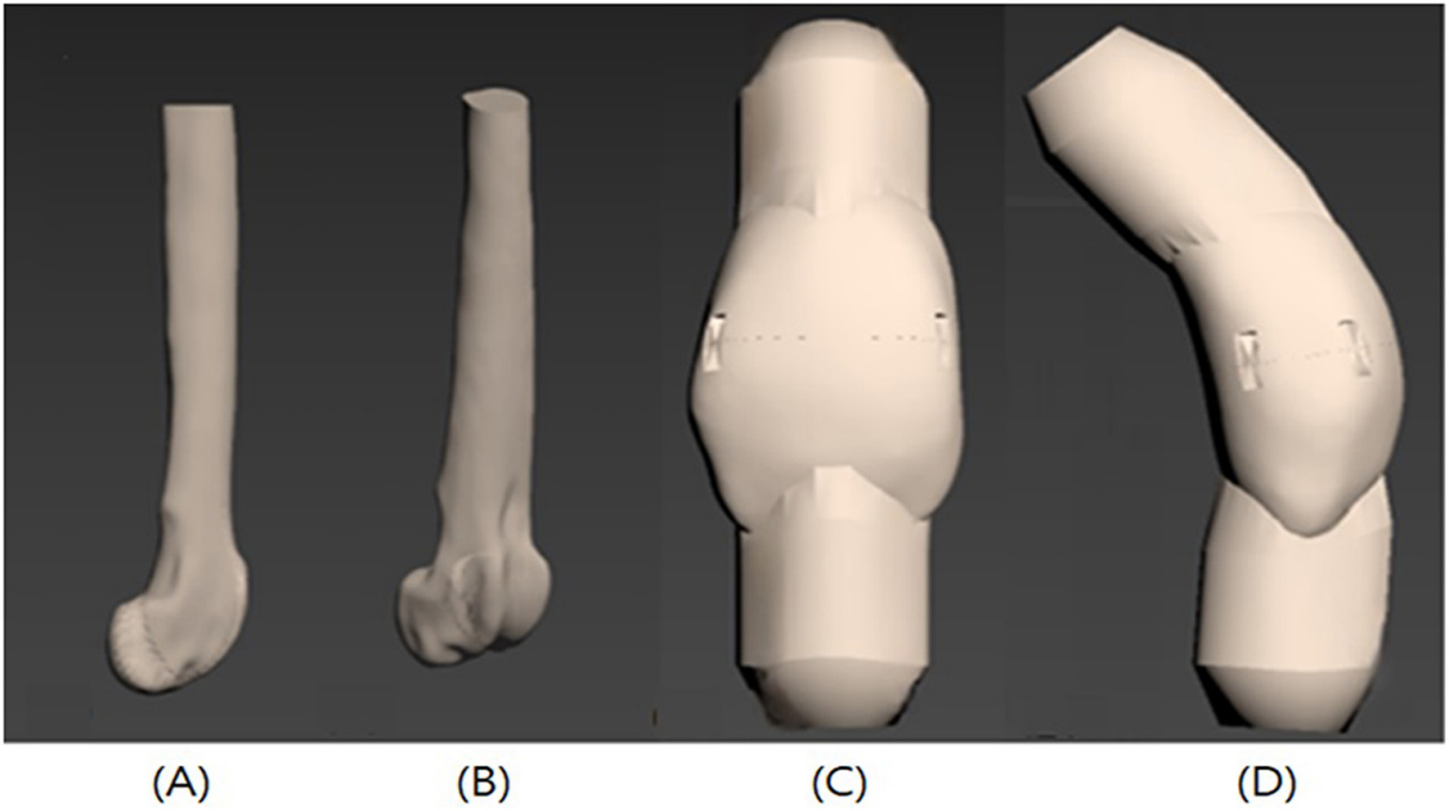
Illustrations of the 3D-printed femur and patella bone model. Femur and Patella with jig for fixation screw is printed with the bone model. **(A)** Femur lateral view, **(B)** Femur craniolateral view, **(C)** Patella cranial view, **(D)** Patella lateral view.

The bone model was printed from the proximal to the distal two-thirds of the right femur using 3D printing. The patella was also printed by two parts cutting at the midpoint to making a transverse fracture. The upper and lower segments of the patella were printed separately, and protruding structures were designed at both ends where the ligaments attach, allowing insertion of fixation screws (Figures 1A, B).

Polycarbonate (PC) was used in 3D printing due to its previously reported compressive strength, fracture stiffness, and overall biomechanical behavior, which are comparable to those of cortical bone (Figures 1C, D) (20).

In the patellar fracture model, the patella itself (excluding the jig) measured 28.2 mm in height, 16.4 mm in width, and 13.3 mm in thickness. To act tensile forces generated by the quadriceps and patellar ligament, raised structures were designed at both the proximal and distal ends of the patella segments, allowing the insertion of 4 mm-thick nut screws. A metal ring was attached to each screw, and 4 mm steel wire ropes were connected to act the tensile force exerted during quadriceps contraction and knee extension.

The femur model was securely fixed onto a wooden plate, and the patella was precisely seated within the trochlear groove. The angle between the upper and lower fixation screw with steel wire rope was set to 135°, reflecting the typical biomechanical angle of a standing dog. Additionally, to mimic the natural force vector of the quadriceps, a pulley system was installed at the top of the plate. This setup enabled vertical tension to be applied to the wire ropes while recreating the oblique direction of force generated by the quadriceps during physiological loading.

In this study, transverse patellar fractures were categorized into three groups, with 10 patellar samples assigned to each group, resulting in a total of 30 samples for testing. Group 1 was fixed by inserting 1.2 mm Kirschner wires bilaterally through the patella, followed by fixation using a 1.0 mm cerclage wire in a figure-of-eight configuration. Groups 2 and 3 were each fixed using only a locking plate and locking screws, utilizing two-hole and four-hole (rectangular configuration) locking plates with a thickness of 1.3 mm, respectively.

### Surgical procedures

#### Group 1: pins and figure-of-eight tension band wire (pin/TBW)

**Figure 2:**
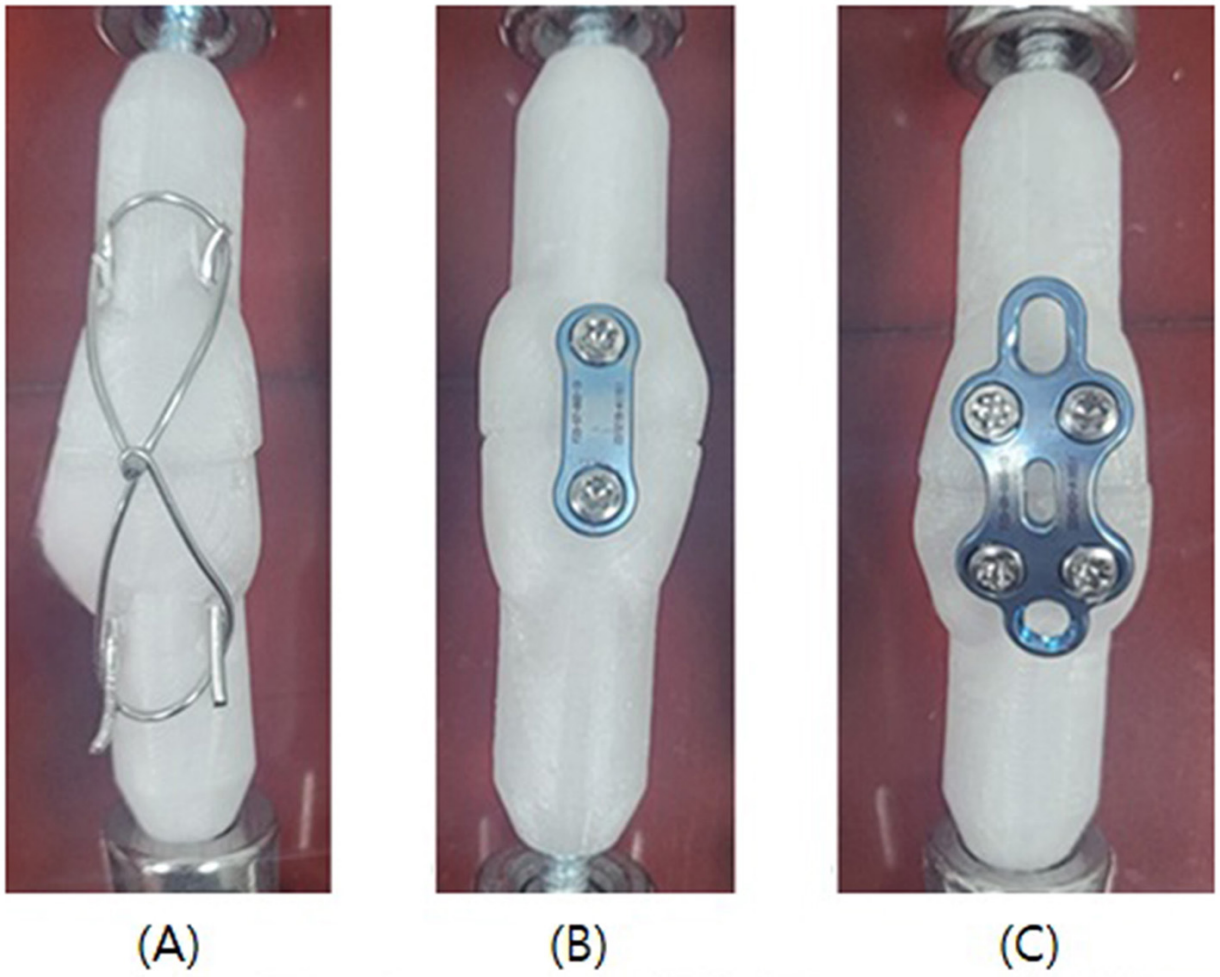
Illustrations of fixation methods. **(A)** Group 1, Two 1.2 mm pins were inserted bilaterally and 1.0 mm cerclage wire was applied figure-of-eight pattern. **(B)** Group 2, A 2-Hole locking plate was contoured and applied for fixation. **(C)** Group 3, A 4-Hole locking plate was contoured and applied for fixation.

In Group 1, the pin/TBW technique was applied (Figure 2A). A 1.2 mm diameter Kirschner wire (Hankiltech Medical Corp., Hwaseong-si, Gyeonggi-do, Republic of Korea) was drilled in a normograde manner, perpendicular to the transverse plane, from either the left or right side of the cut surface of the proximal patellar fragment toward the distal fragment. Following this, the proximal and distal fragments of the patella were anatomically reduced using a pinpoint reduction forcep. A second Kirschner wire was then inserted on the opposite side to achieve bilateral fixation with two pins. Subsequently, a 1.0 mm diameter orthopedic wire (Topmedical Inc., Paju-si, Gyeonggi-do, Republic of Korea) was used to perform a figure-of-eight wiring. The wire was passed behind the proximal and distal ends of the Kirschner wires, tightened firmly, and the ends were twisted three to four times before being cut. The free ends of the Kirschner wires were bent to secure the construct.

#### Group 2: locking plate and locking screw (2-hole)

In Group 2, an anterior locking plate [Human Foot Lock Plate, two-hole type] (Jeil Medical Corp., Mapo-gu, Seoul, Republic of Korea) was used (Figure 2B). Its thickness was 1.3 mm and screw head size was Ø2.3 mm. The upper and lower fragments of the patella were anatomically reduced using pointed reduction forceps, followed by temporary stabilization. The two-hole locking plate was pre-contoured using a plate bender to achieve optimal conformity with the anterior surface of the patella. Screw holes were drilled monocortically at the pre-contoured positions using a 1.8 mm drill bit, followed by insertion of 2.3 mm locking screws.

#### Group 3: locking plate and locking screw (4-hole)

In Group 3, the fracture was repaired using the same procedure as in Group 2, except that a four-hole locking plate was used instead of a two-hole plate (Figure 2C).

### Biomechanical testing

**Figure 3:**
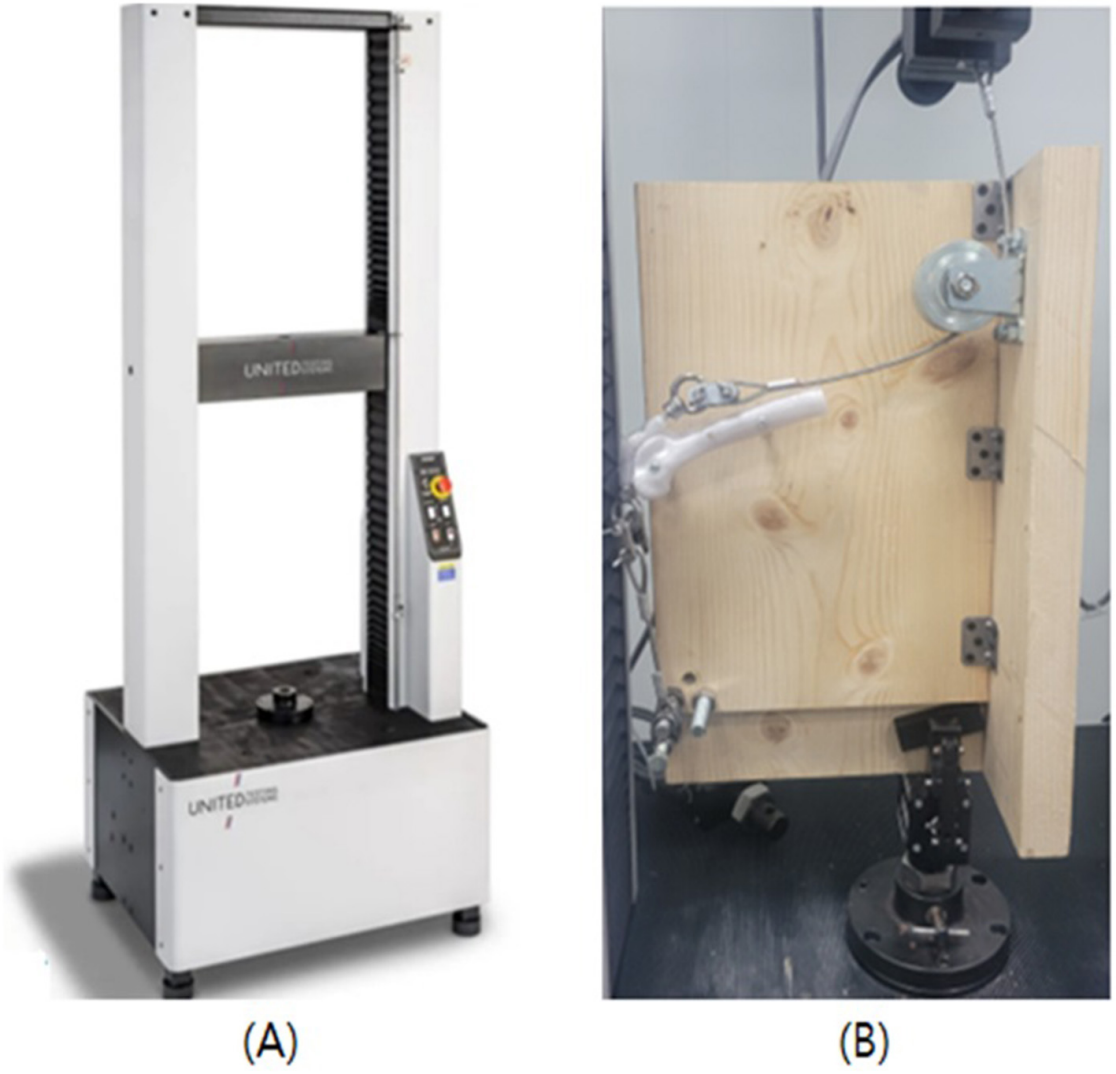
Photographs of universal testing machine and biomechanical testing. **(A)** Experiments were conducted using a universal testing machine. **(B)** Sample was fixed to a jig an angle of 135 degrees.

Biomechanical testing was conducted using a Universal Testing Machine at Daegu Gyeongbuk Institute of Science and Technology (DGIST, Daegu, Korea) (Figure 3A).

All patellar models were positioned within the trochlear groove at a physiological stifle angle of 135°, simulating the normal standing posture of a dog (Figure 3B). The trochlear groove was laterally secured to a wooden support platform. A total of 30 patellar models across three groups underwent biomechanical tensile testing using a Universal Testing Machine (UTM; United SFM-100 kN, United Calibration Corp., Huntington Beach, California, USA). The wooden plate was mounted to the lower grip of the UTM, while the upper grip held the end of a 4 mm steel wire rope. This rope was connected to a nut screw embedded in the proximal end of the patella, acting the tensile force of the quadriceps muscle. Another 4 mm steel wire rope was anchored to the distal part of the patella using a screw to mimic the patellar tendon.

After securing the upper wire rope to the UTM's upper grip, a tensile load was applied at a constant rate of 10 N/min. Tensile forces were recorded at 1, 2, and 3 mm fracture gap distances, as well as at the point of fixation failure.

Fixation failure was defined as a fracture gap exceeding 2 mm between the fracture surfaces. The tensile force at the point of failure was documented. To ensure measurement, the fracture gap was measured using a vernier caliper (Absolute Digimatic Caliper^®^, Mitutoyo Inc., Kanagawa, Japan).

### Statistical analysis

Statistical analyses were performed using SPSS software (Statistical Program for the Social Sciences v.28.0.1.1, SPSS Inc. IBM, Chicago, Illinois. USA). Since each group consisted of only 10 samples, the Kruskal–Wallis test, a non-parametric method, was used to assess statistical significance among groups. A *p*-value of < 0.05 was considered statistically significant. When significant differences were found among groups, *post-hoc* analysis was conducted using the Mann–Whitney *U* test. In this analysis, a *p*-value of < 0.017 was considered statistically significant.

## Results

### Load at 1 mm, 2 mm, 3 mm displacement

In all pin/TBW models (Group 1), the gap between the patellar fracture fragments was measured at displacements of 1, 2, and 3 mm, respectively. When displacement exceeded 3 mm, the fixation was considered to have failed, and no further tensile force measurements were conducted. The mean tensile forces recorded at 1, 2, and 3 mm of displacement in Group 1 were 226.4 ± 26.26, 280.8 ± 27.73, and 342.7 ± 26.98 N, respectively. In Group 2, which employed a two-hole locking plate, only two out of 10 specimens exhibited a 1 mm gap between fracture fragments; the remaining eight specimens demonstrated no measurable displacement up to the maximum tensile force applied. In the two specimens where a 1 mm gap was observed, displacement occurred at an average tensile force of 394.5 N, and plate bending was noted in these cases. In Group 3, where a four-hole locking plate was applied, none of the 10 specimens exhibited any measurable displacement of even 1 mm (Table 1). Among the specimens in the plate groups (Groups 2 and 3), fixation failure was not observed in any specimens except for the two cases in Group 2 where displacement was recorded.

**Table 1:** Mean ± SD for the load at 1, 2, 3 mm displacement and failure for each group.

**Displacement distances**	**Tensile force (N) at displacement (mean** ±**SD)**		
	**Group 1 (*****n*** = **10)**	**Group 2 (*****n*** = **10)**	**Group 3 (*****n*** = **10)**
1 mm	226.4 ± 26.2^a^	394.5^b^ (*n* = 2)	–
2 mm	280.8 ± 27.7^a^	–	–
3 mm	342.7 ± 27.0^a^	–	–
Maximum force	391 ± 31.1^a^	505.6 ± 66.6^b^	556.9 ± 39.0^b^

N, Newton; SD, standard deviation.

Statistically different groups were denoted by different alphabets with superscripts (*p* value < 0.05). When sharing common superscripts, they are not statistically significantly different.

Thus, in order to statistically compare the fixation strength, the maximum tensile failure loads of Groups 2 and 3 were compared with the tensile forces recorded at 1, 2, and 3 mm displacement in Group 1, using the Kruskal–Wallis non-parametric test. The results revealed statistically significant differences among the groups, with a *p*-value of 0.000026 (*p* < 0.05). Subsequent Mann–Whitney *U* tests were conducted as *post-hoc* analyses. Significant differences were found between the 2 and 3 mm displacement conditions of Group 1 and Groups 2 and 3, respectively (*p* = 0.000011), confirming statistical significance at the adjusted threshold (*p* < 0.017). However, no statistically significant difference was observed between Groups 2 and 3 (*p* = 0.52) (Table 2).

**Table 2:** Types of failure for each group.

**Groups**	**Failure types**	**Frequency**
Group 1 (*n* = 10)	Cerclage wire enlongation and pulling out	9
	Cerclage wire breakage	1
Group 2 (*n* = 10)	Breakage of distal fragment on fixation screw	10
Group 3 (*n* = 10)	Breakage of distal fragment on fixation screw	10

Types of failure were recorded after each experiment.

### Maximum tensile force

**Figure 4:**
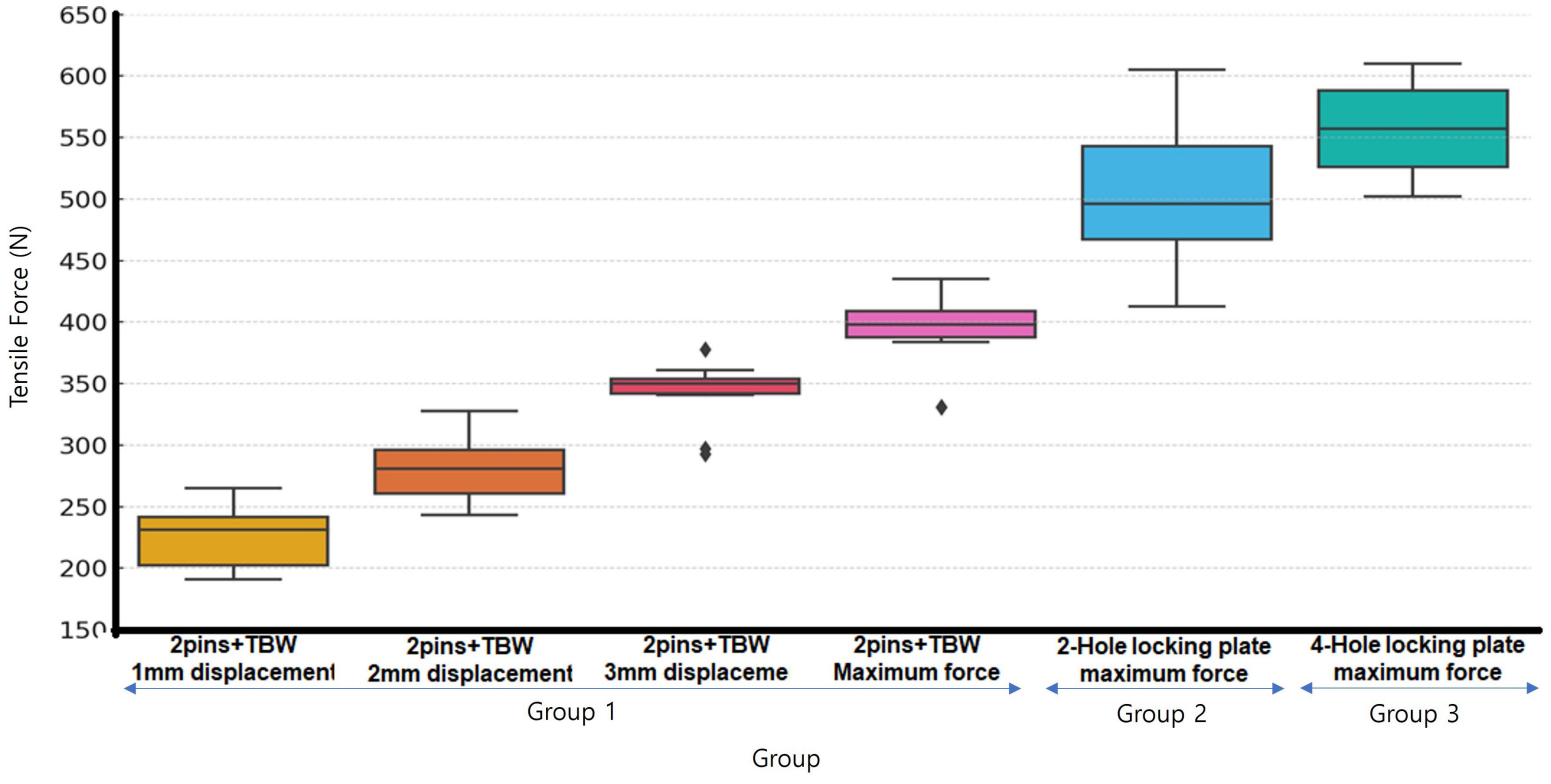
Box-Whisker plot of loads for each group at 1, 2, 3 mm displacement and Maximum failure load. All pin/TBW groups exhibited displacement and failure at lower forces compared to all plate groups. No statistically significant difference was observed between the 2-hole and 4-hole plate groups.

The mean maximum tensile failure load measured in all Group A specimens was 391 ± 31.1 N. In Group B, the mean maximum tensile failure load was 505.6 ± 66.64 N, while in Group C, it was measured at 556.9 ± 39.63 N (Table 1). Significant differences were observed among Group 1, Group 2, and Group 3 (Figure 4).

### Mode of failure

In Group 1, nine out of 10 specimens exhibited fixation failure characterized by elongation of the cerclage wire followed by its detachment from the Kirschner wire. In the remaining one specimen, failure occurred due to rupture of the cerclage wire. In Groups 2 and 3, all specimens showed fractures occurring at the protrusion where the fixation screw for the steel wire rope was inserted into the distal part of the patella.

## Discussion

This study was conducted to biomechanically compare the fixation method using a commercially available locking plate alone with the conventional pin/TBW technique in a canine patellar transverse fracture model reproduced using 3D printing technology. The results demonstrated that the locking plate alone provided superior mechanical stability compared to the pin/TBW method, suggesting that this technique may serve as a robust alternative to traditional fixation methods (21).

The use of plate fixation for patellar fractures has been rarely reported in the field of veterinary medicine. Accordingly, the present study was designed with reference to biomechanical studies in human medicine that evaluated the efficacy of similar fixation techniques. In most biomechanical studies reported in the human literature, fixation failure is defined as an increase in the fracture gap exceeding 2 mm (22–24). This threshold is based on the correlation between postoperative fracture gap widening and an increased risk of osteoarthritis development as well as poor clinical outcomes, with 2–3 mm commonly adopted as the critical limit (25–27).

Various surgical techniques for patellar fractures used in human medicine are predominantly evaluated based on passive range of motion of the knee joint, reflecting biomechanical loading conditions typically encountered during the early postoperative rehabilitation period-namely, non-weight-bearing with the use of crutches. In contrast, dogs generally exhibit spontaneous weight-bearing immediately after anesthesia and surgery, even when bandages or splints are applied. Therefore, applying human-based experimental conditions directly to veterinary clinical scenarios may not be appropriate.

Based on the biomechanical characteristics of dogs, ~30% of the body weight is borne by the hindlimbs during walking (28), and this vertical ground reaction force increases to 76%−107% depending on the breed during trotting (29). In the case of the 45 kg Akita used in this study, the estimated load on the hindlimb ranges from ~335.16 to 471.90 N. Although the exact tensile force acting on the patella and patellar ligament has not been clearly identified, it has been reported that during the stance phase of gait, the quadriceps muscle exerts a load equivalent to ~94.8% of the body weight (30), corresponding to about 418.1 N under the current experimental conditions.

In the pin/TBW fixation group of this study, an increase in fracture gap >2 mm was observed under a mean tensile load of 280.8 N, and displacement >3 mm occurred at 342.7 N. These findings suggest that the pin/TBW technique may result in gap widening or fixation failure even under the lower range of physiological loading during trotting.

In contrast, the two locking plate models evaluated in this study maintained a fracture gap of < 1 mm even under tensile forces exceeding 500 N. These constructs demonstrated superior biomechanical stability, withstanding loads greater than those exerted by the quadriceps during trotting and the stance phase, indicating a more reliable fixation compared to the conventional pin/TBW technique.

According to previous study on patellar fracture repair, the application of the pin/TBW technique has generally resulted in the formation of a fracture gap exceeding 2 mm or fixation failure due to implant failure under quadriceps tensile forces below 500 N (18). There are consistent with the results of the present study. Furthermore, the average tensile forces associated with 1–3 mm of displacement in this study were slightly higher than those reported in previous literature. This discrepancy is presumed to be due to variations in the diameter and number of Kirschner wires and cerclage wires used for fracture fixation, which may have allowed the displacements to occur under higher quadriceps tensile forces (31–33).

In this study, most of the locking plate models maintained a fracture gap of < 1 mm, yet fractures consistently occurred at the screw insertion site in the distal patellar segment. This failure pattern is presumed to result from localized structural weakening due to reduced print density in the region where space was allocated for screw insertion at the patellar ligament attachment site during the 3D printing process.

As previously reported in the literature, maintaining a fracture gap of < 2 mm under tensile loading conditions is considered a clinically important factor for optimal fracture healing and rehabilitation. Based on the findings of this study, the locking plate fixation method demonstrated superior mechanical stability compared to the structural strength of the bone model itself. Therefore, it is anticipated that in clinical applications, this fixation method would maintain stability even under higher tensile forces.

In human medicine, the pin/TBW technique is the most commonly used surgical method for patellar fractures and serves as the reference standard in most biomechanical evaluations comparing newly developed fixation techniques (34–37).

Recently, numerous studies have been conducted using cadaveric specimens and clinical patients to compare the tensile strength, cyclic loading performance, postoperative complications, and patient discomfort between locking plates and the pin/TBW technique. These studies have consistently reported that locking plate fixation offers superior mechanical stability and improved clinical outcomes compared to the conventional pin/TBW method (19, 38). The results of the present study also demonstrated similar trends to those observed in human medicine, suggesting that locking plate fixation may provide significant biomechanical advantages in veterinary applications as well.

In human medicine, patella-specific locking plates are commercially available in various configurations. Depending on the fracture pattern, these plates are used either alone or in combination with other fixation techniques such as cannulated screws, cerclage wires, fiber wires, and Kirschner wires to achieve stable reconstruction of patellar fractures (19, 38). This multimodal fixation approach allows for application not only in simple transverse fractures but also in more complex fracture types.

Furthermore, there have been clinical case reports in which locking plates were successfully used in revision surgeries following failure of the conventional pin/TBW technique. These findings suggest that locking plate fixation provides superior mechanical stability and enables more effective restoration of the anatomical structure of the patella. As a result, it may offer significant clinical advantages by facilitating faster rehabilitation and earlier return to normal activity (39).

To the best of the author's knowledge, only one case has been reported to date in which a locking plate was used as a sole fixation method for patellar fracture repair in dogs. In the referenced study, cadaveric patellae were reconstructed using a 2.6 mm thick plastic plate with a total of eight threaded Kirschner wires which was inserted four into each of the proximal and distal patellar fragments. The results demonstrated superior fixation strength under both tensile loading and cyclic loading conditions compared to the conventional pin/TBW technique, which is consistent with the biomechanical stability observed in the present study (18).

In addition, the previous study employed monocortical locking screws rather than bicortical screws, due to the risk of damaging the trochlear cartilage and patellofemoral joint when using the latter. Based on the same biomechanical consideration, the present study also adopted the monocortical locking screw technique (40).

Furthermore, in previous study, fixation failure was observed in seven out of 10 specimens treated with the pin/TBW technique (18). The authors attributed this failure to the interposition of soft tissue between the patella and the implants (Kirschner wires and cerclage wire), which may have caused gaps and loosening during wire tightening, ultimately compromising bone-to-implant contact (24, 34, 35, 37, 41–43). In contrast, the present study used 3D-printed bone models without soft tissue, allowing the metal implants to be directly secured to the bone surface. However, this distinction suggests that *in vivo* application of the pin/TBW technique may result in fracture gap formation of 1, 2, or even 3 mm under loading conditions lower than the tensile forces observed in the present study.

Among the specimens fixed with a two-hole locking plate, two samples exhibited a 1 mm fracture gap displacement. In specimens where fixation failure occurred at maximum tensile load, visible structural deformation was observed, particularly in the upper patellar fragment where the plate hole showed bending along the direction of the applied tensile force. This suggests that when tensile force from the quadriceps femoris is applied obliquely, the expected compression force between the patella and trochlear groove may not be effectively transmitted to the upper fragment. Such a phenomenon is likely due to the absence of soft tissue structures, which play a role in maintaining compressive stability within the joint.

In contrast, no such deformation was observed in the specimens with the four-hole locking plate. This may be attributed to the greater surface area of the four-hole plate, which likely provided enhanced structural stability under the same loading conditions. These findings indicate that the four-hole locking plate offers superior biomechanical resistance to strong tensile forces generated by the quadriceps, and may result in a lower rate of fixation failure.

This study aimed to replicate the stifle joint using 3D printing technology; however, soft tissue structures such as the joint capsule and fascia could not be reproduced, with only the patella and femur included in the model. This limitation prevented full replication of the *in vivo* environment and posed challenges in simulating compressive forces and biomechanical influences exerted by soft tissues such as the joint capsule and parapatellar ligaments.

The construction of the transverse patellar fracture model used in this study may be considered one of its limitations. The fracture surfaces were fabricated using 3D printing technology, resulting in smooth and standardized interfaces. However, such clean and uniform fracture patterns are rarely encountered in clinical settings. Therefore, it is difficult to determine whether the plates used in this study would produce comparable results if applied to actual clinical cases of transverse patellar fractures.

Additionally, tensile force measurements were performed using a universal testing machine (UTM), while fracture gap displacement was assessed visually using a vernier caliper rather than digital sensors. As a result, there may have been limitations in detecting subtle, real-time changes in fracture gap width during the application of tensile load, potentially introducing measurement error. To minimize this limitation, the tensile loading rate was set as low as possible, and all measurements were performed by a single evaluator to ensure consistency.

Furthermore, the morphology and size of the patella and fracture fragments may vary depending on individual canine patients, and the surgical technique applied in this study may not be universally applicable to all cases. Notably, the locking plate used in this experiment which was selected from among the smallest available in human orthopedics was applied to a large-breed dog weighing 45 kg. However, it may limit the generalizability of locking plate fixation in small-breed dogs commonly kept in Korea. Nevertheless, no statistically significant difference in fixation strength was observed between the two-hole and four-hole locking plates. This finding suggests that the use of smaller two-hole locking plates may also be feasible in small-breed dogs, warranting further investigation through follow-up studies.

## Conclusion

In this study, the application of a commercially available locking plate fixation method for canine transverse patellar fractures demonstrated superior mechanical stability compared to the conventional pin/TBW technique. These findings suggest that locking plate fixation may serve as a more robust and reliable surgical option for the treatment of transverse patellar fractures in clinical veterinary practice. No statistically significant difference in fixation strength was observed between models fixed with a two-hole plate and those fixed with a four-hole plate.

## Data Availability

The original contributions presented in the study are included in the article/supplementary material, further inquiries can be directed to the corresponding author.
